# S9, a Novel Anticancer Agent, Exerts Its Anti-Proliferative Activity by Interfering with Both PI3K-Akt-mTOR Signaling and Microtubule Cytoskeleton

**DOI:** 10.1371/journal.pone.0004881

**Published:** 2009-03-18

**Authors:** Chao Zhang, Na Yang, Chun-hao Yang, Hua-sheng Ding, Cheng Luo, Yu Zhang, Mao-jiang Wu, Xiong-wen Zhang, Xu Shen, Hua-liang Jiang, Ling-hua Meng, Jian Ding

**Affiliations:** 1 Division of Anti-Tumor Pharmacology, State Key Laboratory of Drug Research, Shanghai Institute of Materia Medica, Chinese Academy of Sciences, Shanghai, China; 2 Division of Organic Synthesis, Shanghai Institute of Materia Medica, Chinese Academy of Sciences, Shanghai, China; 3 Drug Discovery and Design Center, Shanghai Institute of Materia Medica, Chinese Academy of Sciences, Shanghai, China; 4 School of Pharmacy, East China University of Science and Technology, Shanghai, China; Western Illinois University, United States of America

## Abstract

**Background:**

Deregulation of the phosphatidylinositol 3-kinases (PI3K)/Akt/mammalian target of rapamycin (mTOR) pathway plays a central role in tumor formation and progression, providing validated targets for cancer therapy. S9, a hybrid of α-methylene-γ-lactone and 2-phenyl indole compound, possessed potent activity against this pathway.

**Methodology/Principal Findings:**

Effects of S9 on PI3K-Akt-mTOR pathway were determined by Western blot, immunofluorescence staining and in vitro kinas assay. The interactions between tubulin and S9 were investigated by polymerization assay, CD, and SPR assay. The potential binding modes between S9 and PI3K, mTOR or tubulin were analyzed by molecular modeling. Anti-tumor activity of S9 was evaluated in tumor cells and in nude mice bearing human cancer xenografts. S9 abrogated EGF-activated PI3K-Akt-mTOR signaling cascade and Akt translocation to cellular membrane in human tumor cells. S9 possessed inhibitory activity against both PI3K and mTOR with little effect on other tested 30 kinases. S9 also completely impeded hyper-phosphorylation of Akt as a feedback of inhibition of mTOR by rapamycin. S9 unexpectedly arrested cells in M phase other than G1 phase, which was distinct from compounds targeting PI3K-Akt-mTOR pathway. Further study revealed that S9 inhibited tubulin polymerization via binding to colchicine-binding site of tubulin and resulted in microtubule disturbance. Molecular modeling indicated that S9 could potentially bind to the kinase domains of PI3K p110α subunit and mTOR, and shared similar hydrophobic interactions with colchicines in the complex with tubulin. Moreover, S9 induced rapid apoptosis in tumor cell, which might reflect a synergistic cooperation between blockade of both PI3-Akt-mTOR signaling and tubulin cytoskeleton. Finally, S9 displayed potent antiproliferative activity in a panel of tumor cells originated from different tissue types including drug-resistant cells and in nude mice bearing human tumor xenografts.

**Conclusions/Significance:**

Taken together, S9 targets both PI3K-Akt-mTOR signaling and microtubule cytoskeleton, which combinatorially contributes its antitumor activity and provides new clues for anticancer drug design and development.

## Introduction

Phosphatidylinositol 3-kinase (PI3K) plays a central role in a complex, multi-armed signaling network that orchestrates cell response including cell survival, growth, proliferation, angiogenesis, migration and glucose metabolism [1], [2]. PI3K is presumed to activate most of its downstream targets via the Akt protein, which phosphorylates diversified downstream substrates including the mammalian target of rapamycin (mTOR) [2], a master regulator of protein translation. Moreover, PI3K and its downstream components cross-talk with a number of other pathways, such as the Raf/mitogen-activated protein kinase kinase/extracellular signal–regulated kinase (MEK/ERK)–activated pathways [3]. PI3K-mediated signaling network is important in tumorigenesis and progression, which is frequently hyper-activated in a variety of human cancers, as a result of gain-of-function in PI3K, inactivation of the lipid phosphatase PTEN or deregulated growth factor signaling [1], [3], [4]. As such, the PI3K-Akt-mTOR pathway has been validated as a tumor therapeutic target of increasing interests.

Multiple efforts are under way in academia and industry to develop clinically relevant inhibitors of this signaling pathway. Temsirolimus (Torisel®, Wyeth pharmaceuticals), an analog of rapamycin targeting mTOR [5], represents the most successful developed compounds targeting this pathway, which has been approved for the treatment of advanced kidney cancer. Two other rapamycin derivatives RAD-001[6] and AP-23573 are currently in phase III clinical trials. With an increasing understanding of the feedback loops exiting in the PI3K-Akt-mTOR pathway, it has been recognized that inhibition on one output costs at the expense of activation of the others. For instance, rapamycin monotherapy activates Akt through inhibition of an mTOR-dependent retrograde signal [7]. For this reason, there is a growing consensus that the inhibition of combined components in the PI3K-Akt-mTOR pathway likely reflects a mechanistic rationale for the therapeutic options. As a matter of fact, many of the most effective PI3K-Akt-mTOR-targeted cancer therapies owe their activity to inhibition of dual or multiple components in this pathway, such as NVP-BEZ235 (a dual pan-PI3K/mTOR inhibitor) and SF-1126 (inhibitor of all isoforms of PI3K and other PIKK family members), which are now entering into Phase I/II clinical trials [4].

Given the severity of most cancers and their high unmet clinical need, together with the fact that the PI3K-Akt-mTOR axis represents an important emerging class of drug targets, we designed and synthesized a series of hybrids of α-methylene-γ-lactones and 2-phenyl indoles with the aim of discovery of new inhibitor(s) targeting PI3K-Akt-mTOR pathway [8]. Of them, S9 stood out by functioning as a dual PI3K and mTOR inhibitor and also a microtubule destabilizer. The converged outcome of both cytostatic and cytotoxic identities of S9, together with its appreciable *in vivo* antitumor profiles, help prove to be a promising novel chemical template for the development of axis inhibitors.

## Results

### S9 inhibits PI3k-Akt-mTOR signaling

#### S9 restrains PI3k-Akt-mTOR signaling stimulated by EGF in cancer cells

**Figure 1:**
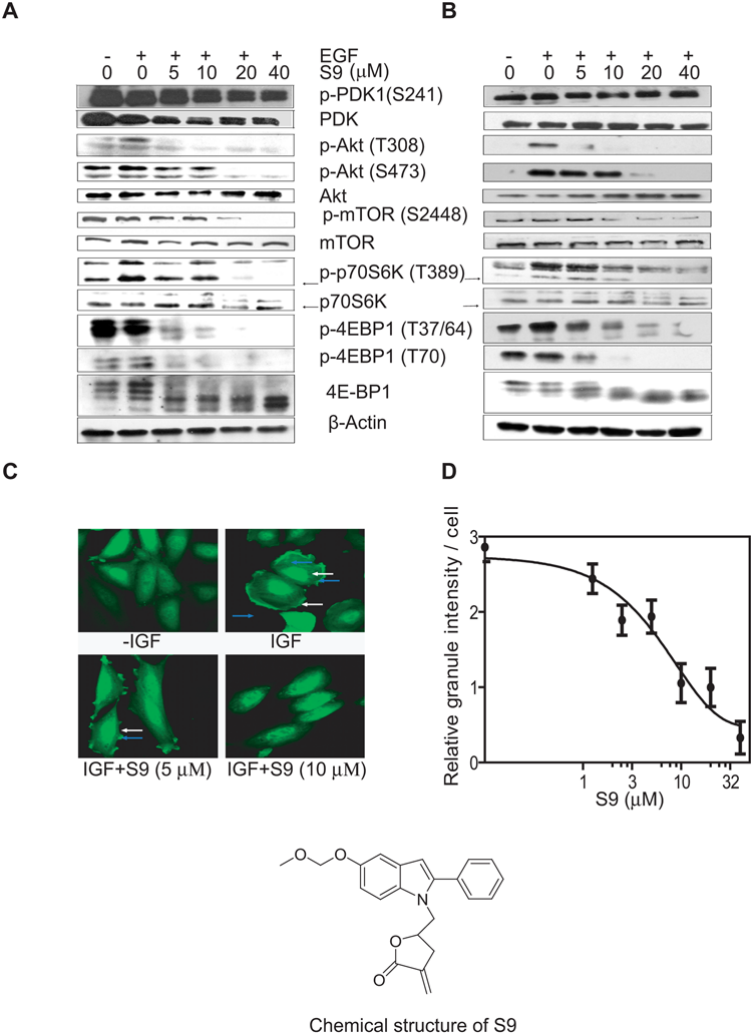
S9 inhibits PI3K-Akt-mTOR signaling. A–B), S9 depresses EGF-triggered activation of PI3K-Akt-mTOR signaling pathway. Serum-deprived Rh30 cells (A) and SK-OV-3 cells (B) were treated with indicated concentrations of S9 for 1 h followed by EGF (50 ng/ml) stimulation for 10 min. Cells were harvested for Western blot analysis with antibodies specific for p-PDK1 (S241), PDK, p-Akt (T308), p-Akt (S473), Akt, p-mTOR (Ser2448), mTOR, p-p70S6K (T389), p70S6K, p-4E-BP1(T37/64), p-4E-BP1 (T70), 4E-BP1 and actin. Arrows indicate p70 isoform of S6 kinase protein. C) S9 blocks Akt membrane translocation and membrane ruffling. CHO (pCORON1000-EGFP-Akt) cells seeded on chamber were starved for 2 h then treated with 5 or 10 µM S9 for 1 h followed by IGF stimulation for 5 min. Fluorescent pictures were captured with confocal fluorescent microscopy. White arrows indicate cell membrane ruffling. Blue arrows indicate fluorescent foci. D) Total Akt granule intensity in each CHO (pCORON1000-EGFP-Akt) cell was counted with statistic module by IN Cell Analyzer 1000. Data shown are representative from two independent experiments.

We have previously reported that S9 potently down-regulated phosphorylation of Akt, mTOR, p70S6K and 4E-BP1 upon stimulation with EGF in Rh30 cells [8]. To gain insight of the effect of S9 on the PI3k-Akt-mTOR signaling, Rh30 cells were exposed to various concentrations of S9 followed by EGF stimulation. Although located in the upstream of this pathway, phosphorylation of PDK-1 at Ser 241 was insensitive to serum deprivation or EGF stimulation (Fig 1A), which is consistent with the observation reported elsewhere [9]. Phosphorylation of PDK-1 was slightly depressed in the presence of 40 µM S9. In contrast, S9 significantly inhibited phosphorylation of Akt at both Ser 473 and Thr 308 (Fig 1A). Compared with Ser473, Thr308 phosphorylation was more vulnerable to S9 treatment, which might be due to phosphorylation of Ser 473 prior to the phosphorylation at Thr 308 and is important for the recognition and activation of Akt by PDK-1 [10]. Similarly, S9 abrogated phosphorylation of mTOR at Ser 2448 and its downstream proteins p70S6K at Thr 389 and 4E-BP1 at Thr 37/64 and Thr 70 in a concentration-dependent manner. The inhibitory activity of S9 on the PI3K-Akt-mTOR signaling was also confirmed using ovary carcinoma SKOV-3 cells (Fig 1B). Inactivation of the PI3K-Akt-mTOR pathway was also reflected by decrease in the kinase activities of PI3K, Akt and mTOR in the RH30 cells after S9 treatment (Fig S1).

#### S9 blocks Akt cellular translocation and cell membrane ruffling

When cell is stimulated with growth factor, such as insulin, insulin-like growth factor (IGF) and EGF, Akt aggregates around cell membrane responding to recruitment by PIP3, which was a catalytic product of activated PI3K. Activation of PI3K also induces cell membrane ruffling [11]. Once the activity of PI3K is harshly prohibited, aggregation of Akt and membrane ruffling disappears even under growth factor stimulation. As shown in Figure 1C, addition of IGF triggered Akt to aggregate on membrane in EGFP-Akt-stably-transfected CHO cells deprived of serum, demonstrated as highly fluorescent foci near the membrane (blue arrow). Membrane ruffling (white arrow) was also observed in these cells after IGF treatment. However, exposure of cells to S9 before IGF stimulation abrogated Akt translocation and membrane ruffling (Fig. 1C). The granule intensity in each cell was measured and statistically analyzed by IN Cell Analyzer workstation version 3.2. As shown in Figure 1D, S9 reduced the intensity of Akt granule in a concentration-dependent manner. S9 also inhibited PI3K activity and block membrane ruffling in Rh30 cells (Fig S2).

#### S9 inhibits the catalytic activities of PI3K and mTOR

**Figure 2:**
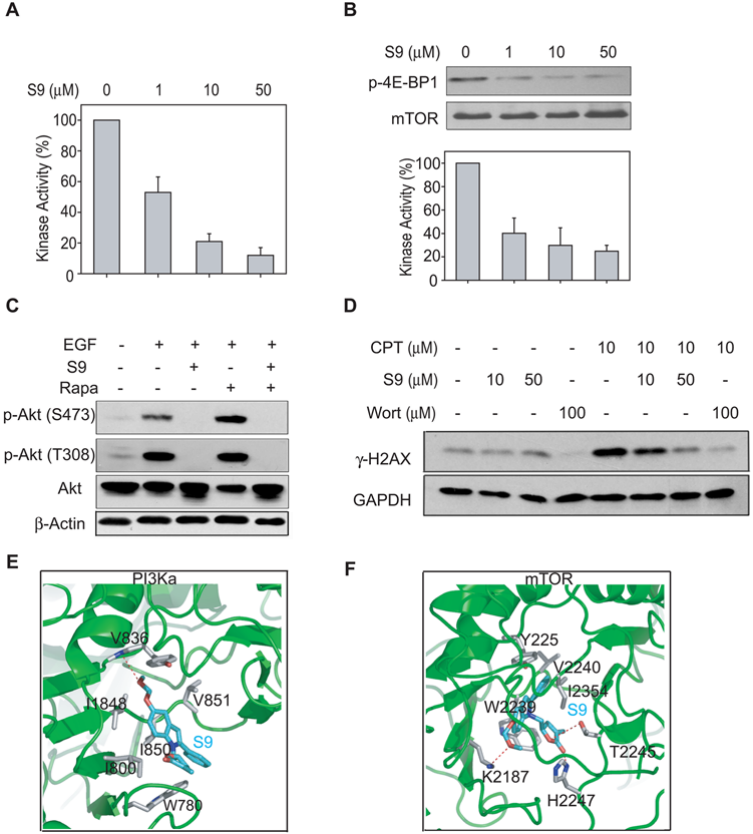
S9 inhibits the kinase catalytic activity of PI3K and mTOR. A–B) Active kinases were immunoprecipitated from exponentially growing Rh30 cells and kinase assays were performed. In the presence of indicated concentrations of S9, phosphorylation PIP2 to PIP3 were measured by ELISA (A). Phosphorylation of 4E-BP1 (B) was detected by Western blot. Band intensity was quantitated by optical densitometric analysis and normalized to vehicle control. Representative images were presented and relative kinase activity was plotted as mean±SD of three independent experiments. C) S9 abrogates rapamycin-induced hyperphosphorylation of Akt (Ser 473). Rh30 cells were starved overnight and treated with 10 µM of S9 and/or rapamycin (Rapa) for 1.5 h on the next day, followed by stimulation with 50 ng/ml EGF for 10 min. Phosphorylated Akt at Serine 473 and Threonine 308 and total Akt were detected by Western blot. D) Rh30 cells were exposed to 10 µM camptothecin (CPT) for 1 h after pre-incubation with indicated concentrations of S9 or wortmannin (Wort) for 30 min. Cells were collected and γ-H2AX levels were detected with Western blot analysis. E–F) The binding mode of S9 within PI3K (E), mTOR (F). The protein is represented by cartoon. The residues interacting with the compounds are shown in sticks. All of the structural diagrams were prepared using PyMOL (http://pymol.sourceforge.net).

As S9 inhibits the PI3K-Akt-mTOR pathway signaling cascade, PI3K kinase assays were first carried out to determine whether S9 inhibits the catalytic activity of PI3K. PI3K were activated with EGF and immunoprecipitated from Rh30 cells, and kinase assays were performed in the presence of S9. As shown in Figure 2A, the ability of PI3K to generate PIP3 decreased after S9 treatment in a concentration-dependent manner and the activity decreased to 53.2% in the presence of 1 µM S9. Since S9 is capable of inhibiting the activity of PI3K, we detected whether S9 inhibits the activity of mTOR, which bears a highly homologous COOH-terminal catalytic domain with PI3K. As shown in Figure 2B, phosphorylation of 4E-BP1 by mTOR was greatly down-regulated by S9 treatment. 1 µM S9 decreased mTOR kinase activity by 58.7%. Thus, S9 is a new dual inhibitor of PI3K and mTOR.

Rapamycin and its derivatives, which specifically inhibit mTOR, induced rapid Akt (Ser473) phosphorylation both in cancer cells and in clinical patient tumors [7], presumably due to inhibition of a mTOR-dependent retrograde signal [12]. This effects evoked impugnation for rapamycin and its derivatives' application in clinical cancer therapy. As shown in Figure 2C, rapamycin treatment induced up-regulation of Akt phosphorylation at Ser 473 in Rh30 cells, with no influence on phosphorylation at Thr 308, which was consistent with previous report [13]. Although S9 is able to inhibit the activity of mTOR, S9 treatment alone does not induce hyper-phosphorylation of Akt at Ser 473 (Fig. 2C). Moreover, co-current treatment with S9 and rapamycin completely attenuated the hyper-phosphorylation of Akt at Ser 473 induced by rapamycin (Fig. 2C), indicating S9 could circumvent activation of Akt triggered by mTOR inhibition.

#### S9 blocks ATM- and ATR-regulated DNA repair pathways

ATM, ATR and DNA-PK, which are involved in DNA damage response, also belong to the phosphoinositide 3-kinase-related kinases (PIKK) family and share highly homologous catalytic domain with PI3K and mTOR. We next determined the effect of S9 on the activities of ATM, ATR and DNA-PK by measuring the level of phosphorylated H2AX (γH2AX). H2AX could be quickly phosphorylated by ATM, ATR and DNA-PK upon DNA strand breaks induced by CPT [14]. As shown in Fig 2D, Rh30 cells displayed a robust increase in γH2AX level after exposure to CPT for 1 h. Co-treatment with S9 significantly abrogated the up-regulation levels of γH2AX at concentrations above 50 µM, indicating that S9 also inhibited the kinase activity of ATM, ATR or/and DNA-PK in Rh30 cells.

To determine the selectivity of S9 on kinases, we tested the effect of S9 on 9 tyrosine kinases and 21 serine/threonine kinases. We found that S9 possessed weak or no inhibition on the tested kinase panel (Table S1), indicating S9 is selective against PI3K and mTOR, and against ATM, ATR and DNA-PK as well.

#### S9 potentially binds to PI3K and mTOR

Based on the facts that most PI3K inhibitors apparently bind to the ATP-binding site of p110, the catalytic subunit of PI3K [15], and that S9 is a pan-inhibitor for PIKK family members, molecular docking approach was performed to predict their potential binding sites and possible binding mode between S9 and p110α (Figure 2E). The docking results indicated that S9 could bind to the ATP pocket with high affinity. For a better resolution, a close-up view of the binding interface was developed. S9 forms one hydrogen-bond with the main-chain of Tyr 836 and hydrophobically interacts with Trp 780, Ile 800, Ile 848, Ile 850 and Val 851 of the enzyme. In addition, overall arrangements of S9 and ATP are very similar comparing their binding modes in the active site of PI3K. Likewise, the S9 hydrogen bonds to the Thr 2245, Lys 2187 and His 2447 of the enzyme in the mTOR-S9 complex (Figure 2F). Among them, Lys2187, which are highly conserved in PIKK family members [16], corresponds to Lys833 in PI3K. These results provide us a clue that S9 may directly target PI3K and mTOR.

### S9 perturbs microtubule skeleton via binding to colchicine-binding sites of tubulin

#### S9 arrests tumor cells in M phase

**Figure 3:**
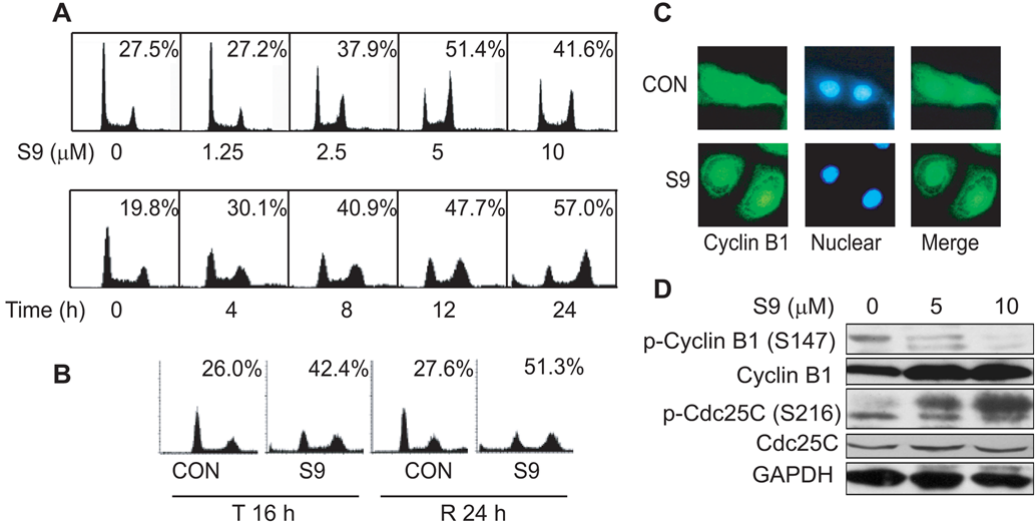
S9 arrests tumor cells in M phase. A) S9 induces does-dependent and time-dependent G2/M phase arrest. SK-OV-3 cells grown in serum-supplemented medium were treated with S9 for 24 h. Cells were collected and subjected to FACS analysis. Data shown are percentage of cell population in G2/M phase. B) S9-induced G2/M phase arrest is irreversible. SK-OV-3 cells were treated with S9 for 16 h (T16), and then S9 was washed out. Cells were further incubated in fresh medium for 24 h (R24). Cells were collected and DNA content was analyzed by FACS analysis. C) Aggregation of cyclin B1 in nucleus after S9 treatment. SK-OV-3 cells were exposed to 5 µM S9 for 24 h. Cells were fixed and stained with anti-cyclinB1 antibody (green) and DAPI (blue). D) S9 decreases the level of phosphorylated cyclin B1 and increases phosphorylation of cdc25C. SK-OV-3 cells were exposed to 5 µM S9 for 24 h and western blot analyses for p-cyclinB1 (Ser 147), total cyclinB1, p-cdc25C (Ser 216) and cdc25C were performed. Glyceraldehyde 3 phosphate dehydrogenase (GAPDH) served as a loading control.

As a common consequence of PI3K-Akt-mTOR signaling inhibition, decreased expression of cyclin D1 will blockade cell cycle transition in G0/G1 phase [17], [18]. To explore the effect of S9 on cell cycle distribution, SKOV-3 cells were treated with indicated concentrations of S9 for 24 h. As shown in Figure 3A, S9 unexpectedly arrested cells in G2/M phase in a concentration-dependent manner up to 5 µM. Cell population in G2/M phase decreased, accompanied with increase in cell population in G1 phase in the presence of 10 µM of S9, which could be a result from PI3K-Akt-mTOR inhibition. S9 also arrested SK-OV-3 cells in G2/M phase time-dependently (Fig. 3A, lower panel). To examine whether the S9-induced G2/M arrest was reversible, SKOV-3 cells were treated with S9 for 16 h (T16) and then S9 was washed out, followed by 24 h (R24) additional incubation in fresh media. We found that the cells arrested in G2/M phase could not re-enter the normal cell cycles progress (Fig. 3B), indicative of irreversible G2/M phase arrest by S9.

To differentiate whether S9 induced G2 phase or M phase arrest, SKOV-3 cells were treated with S9 and stained with anti-Cyclin B1 antibody. Persistent residence of cyclin B1 in nuclei is an indication of cell mitosis block, as well as a marker to distinguish M phase arrest from G2 phase arrest [19]. Most of cylclin B1 aggregated in nuclei after S9 treatment, indicating that S9 induced M phase arrest (Fig 3C). S9 treatment also resulted in decreased phosphorylated cyclin B1 at Ser 147 and increased phosphorylated cdc25c at Ser 216 and total cyclin B1 (Fig 3C), which is in accordance with those agents that induce M phase arrest [20], [21].

#### S9 disturbs cytoskeleton

**Figure 4:**
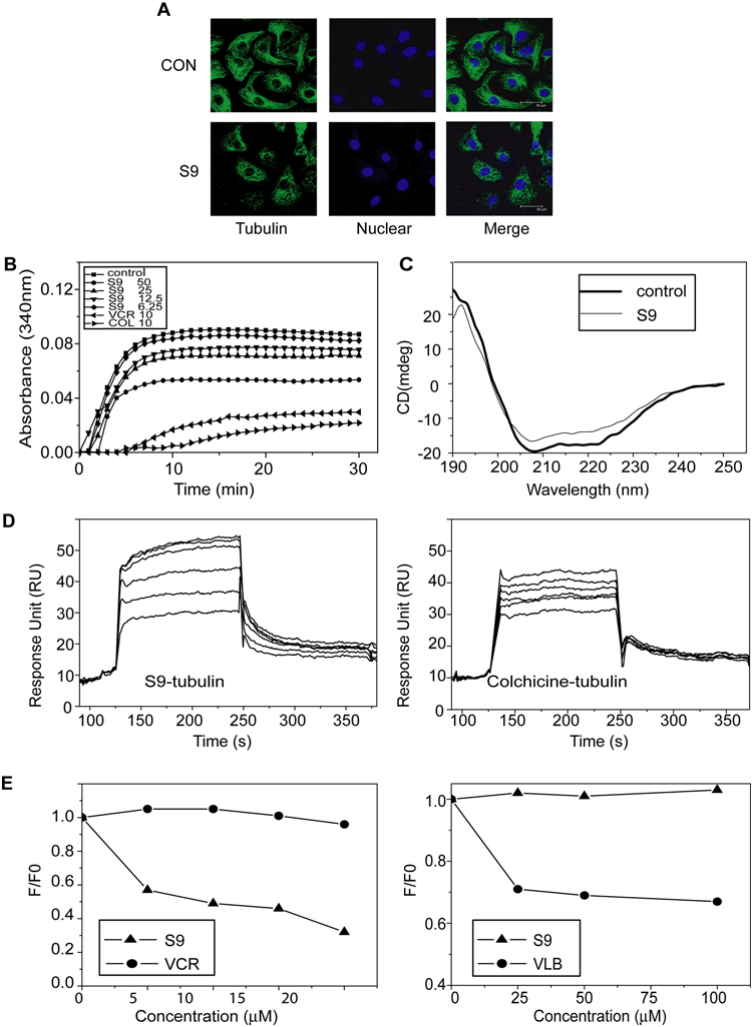
S9 disturbs the microtubular structure by binding to tubulin directly and inhibiting tubulin polymerization. A) S9 disturbs the cell skeleton structure. SK-OV-3 cells were exposed to 5 µM S9 for 24 h and stained with anti-tubulin antibody (green) and DAPI (blue). Scale bar: 30 µm. Data shown are representative of at least three independent experiments. B) Inhibition on purified tubulin polymerization by S9. Purified pork tubulin was incubated with S9, vincristine (VCR) or colchicine (COL) or vehicle in reaction buffer. The optical density (OD) at 340 nm was measured with a spectrophotometer every 1 min till the OD value became stable. C). Disruption secondary structure of tubulin by S9. Purified tubulin and 25 µM S9 were co-incubated in reaction buffer for 1 h. The circular dichroism (CD) spectra were recorded. The characterized absorbance wave lengths were 209/222 nm for α–helix and 196 nm for β–sheet. D) S9 (left panel) and colchicines (right panel) bind to tubulin directly. Purified tubulin was immobilized on sensor chip. Association and dissociation rate constants and real-time binding capacity were recorded by Surface Plasmon Resonance (SPR). The Kd values were computed by Biacore evaluation Version 3.0 software. E) Competitive binding of S9 to colchicine-binding sites on tubulin but not vinblastine-binding sites. Tubulin was co-incubated with indicated concentrations of S9 or vincristine for 1 h, then colchicine was added (left panel). Right panel, tubulin was co-incubated with S9 or vinblastine (VLB) for 1 h followed by addition of BODIPY-FL-vinblastine. The fluorescence was measured by spectrofluorometer. All assays were repeated twice and representative data were shown.

The compounds which arrest cells in M phase are mostly tubulin-targeting agents. We tested whether S9 disrupted the organization of cytoskeleton. The microtubule structure of S9-treated cells was visualized via immunocytochemistry. As shown in Figure 4A, microtubule formed intact network with fine mesh in untreated cells, while in S9-treated cells, tubulin was dispersed as dot, and cells appeared as weaker staining of anti-tubulin antibody which was presumably due to reduction of microtubular materials by depolymerization resulted from S9 treatment (Fig. 4A).

#### S9 inhibits tubulin polymerization in vitro and causes a conformational change in tubulin

As S9 has been shown to disturb microtubule cytoskeleton in SKOV-3 cells, we tested the effects of S9 on tubulin polymerization *in vitro*. The polymerization of purified tubulin at 37°C in the presence of tested compounds or vehicle control was monitored spectrophotometrically. In the absence of compounds, purified tubulin (15 µM) was polymerized in a time-dependent manner. S9 treatment inhibited tubulin polymerization in a concentration-dependent manner (Fig. 4B). The effect of S9 on the second structure of tubulin was monitored by far-UV CD spectroscopy. As shown in Figure 4C, 25 µM S9 induced a secondary structure change of tubulin, characterized by the alteration of α-helix and β-sheet content.

#### S9 directly bind to colchicine-binding site on tubulin

Because S9 inhibits tubulin polymerization and causes conformational change of tubulin, direct binding of S9 to tubulin was next determined using SPR biosensor technology, with a well-characterized tubulin destabilizer colchicine as a positive control. Various concentrations (50.0, 35.0, 24.5, 17.2, 12.0, 8.40, 5.88 µM) of S9 and colchicine were injected for binding with tubulin immobilized on the CM-5 sensor chips. Real time sensor grams were recorded. As shown in Figure 4D, injection of S9 or colchicine caused a significant increase in SPR response in a concentration-dependent manner. General fitting and steady-state binding affinity calculated with Biacore evaluation Version 3.0 software helped release a relative high binding affinity for tubulin with the dissociation constant (Kd) value of 11.1 µM, which was two-fold lower than that of colchicine (24.6 µM).

Most compounds that inhibit tubulin polymerization bind to either colchicine- or vinblastine-binding sites[21]. We investigated whether S9 binds to either of these two sites via competitive binding assays. The intrinsic fluorescence of colchicine increases upon binding to tubulin [22], which could be used as an index for S9 competition with colchicine in tubulin binding. As shown in Figure 4E, left panel, the fluorescence of colchicine-tubulin complex reduced in the presence of S9 in a dose-dependent manner, suggesting that colchicine failed to bind to tubulin-S9 complex. In contrast, the fluorescence remained unchanged when vincristine, a typical vinblastine-binding site drug, was incubated with colchicine and tubulin. A fluorescent analogue of vinblastine (BODIPY FL-vinblastine) was employed to detect whether S9 bound to vinblastine-binding site. S9 had no effect on the fluorescence BODIPY FL-vinblastine while the fluorescence reduced in the presence of vinblastine (Fig. 4E, right panel). These findings provide the evidence that S9 directly binds to colchicine-binding site but not vinblastine-binding site of tubulin.

**Figure 5:**
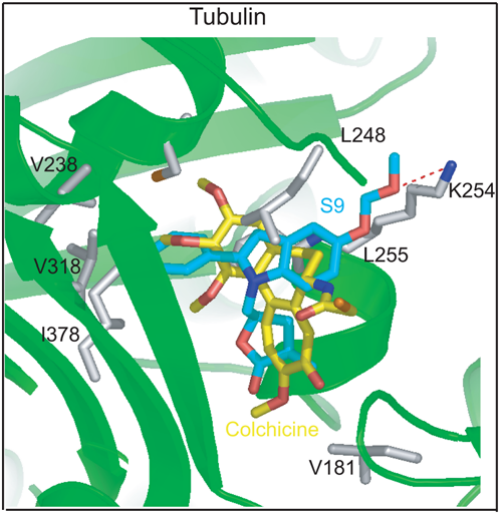
The binding mode of S9 within tubulin. The protein is represented by cartoon. The residues interacting with the compounds are shown in sticks. S9 and colchicine are also represented by sticks and colored in cyan and yellow, respectively. All of the structural diagrams were prepared using PyMOL (http://pymol.sourceforge.net).

The superimposed binding models of S9 and colchicine in tubulin were next performed (Fig. 5). Despite their strikingly difference in structures, the conformation of S9 occupies similar Cartesian space in the colchicine site. Moreover, S9 hydrophobically interacts with Val 181, Cys 241, Val 318, Lys 352 and Ile 378, which interact similarly with colchicine. Of particular note, the MOMO group, lying in the side chain of the phenylindole, formed a hydrogen bond with Lys 254, which might offer a conformation for S9 to place effectively in tubulin. In contrast, S7, in which MOMO group is replaced by H [8], has no effect on tubulin polymerization (data not shown).

### S9 disrupts tumor cell growth in vitro and in vivo

#### S9 induces rapid apoptosis in human cancer cells

**Figure 6:**
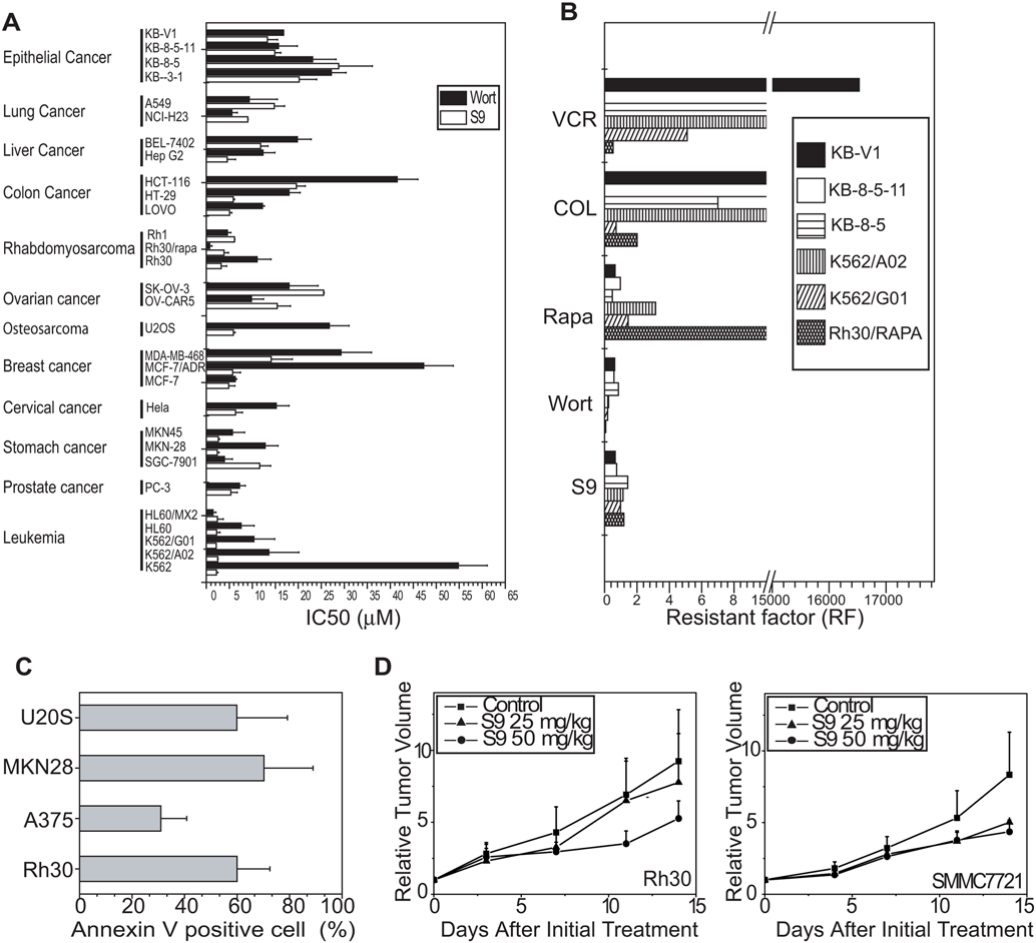
S9 displayed anti-tumor activity in *vitro* and in *vivo* and induces rapid apoptosis in human cancer cells. A) Antiproliferative activity of S9 and wortmannin against a wide panel of tumor cell lines. IC_50_s plotted as mean±SD (µM) were from three separate experiments. B) Resistance factor (RF) of S9 and reference compounds in drug-resistant cancer cells. RF was calculated as the ratio of the IC50 value of the drug-resistant cells to that of the corresponding parental cells. C) S9 induces rapid apoptosis in human cancer cells. Rh30, A375, MKN-28 and U2OS cells were treated with S9 for 8 h. Apoptosis was measured by Annexin V-PI double staining. Apoptosis measured as Annexin-V positive cells were plotted. Data shown were mean±SD from three independent experiments. D) Rh30 (left panel) and SMMC7721 (right panel) xenograft growth inhibition after administration of S9. Rh30 and SMMC7721 nude mice were subcutaneously inoculated with a tumor fragment. The animals were randomly divided in groups when tumor volume reached 100 to 200 mm^3^ and received i.p. injection of normal saline or S9 five times a week for two weeks. Tumor volumes were measured and relative tumor volumes were calculated as tumor volume_Dn_/tumor volume_D0_. Data shown are the mean of six mice.

Because S9 displayed potent inhibition on PI3K-Akt-mTOR pathway as well as tubulin polymerization, the ability of S9 to induce apoptosis was examined in various tumor cells. Cells were subjected to annexin-V-PI doubling staining after exposure to 10 µM S9 for 8 h. As shown in Figure 6C, tumor cells underwent rapid apoptosis after exposure to S9 for 8 h. PI3K inhibitors usually induce G1 phase arrest rather than apoptosis. Rapid apoptosis induced by S9 might represent its combinatorial effect on PI3K-Akt-mTOR pathway and microtubule cytoskeleton.

#### S9 inhibits proliferation of a panel of tumor cells in vitro

The anti-proliferative activity of S9 was measured with SRB assay against a panel of human tumor cells, including oral epidermoid cancer, lung cancer, liver cancer, colon cancer, rhabdomyosarcoma, ovarian cancer, osteosarcoma, breast cancer, cervix cancer, stomach cancer, prostate cancer and leukemia cells. S9 displayed potent activity against the proliferation of a broad spectrum tumor cells with a mean IC_50_ of 9 µM (Fig 6A). Wortmannin, a well-known PI3K inhibitor, was taken as a reference drug with a mean IC_50_ of 16 µM. Although both S9 and wortmannin target PI3K pathway, they displayed different anti-proliferation profiles. For example, S9 were more potent in K562 and U2OS cells than wortmannin. This advantage was also observed in the mini-set of colon cancer cell lines (Fig. 6A). Detailed IC_50_s were shown in Table S2.

The anti-proliferative activity in five pair of MDR cells, Gleevec-resistant K562/G02 and rapamycin resistant Rh30/Rapa cells was also evaluated. Remarkably, S9 demonstrated similar inhibition in drug resistant cancer cells and respective parental cells, with the resistant factors around 1(Fig 6B). Of particular note, S9 remains active against MDR cells unlike other tubulin-interacting drugs such as colchicine and vincristine (Fig 6B).

#### S9 inhibits the growth of human cancer xenografts on nude mice

The anti-tumor activity of S9 in *vivo* was evaluated using human rhabdomyosarcoma Rh30 and human hepatoma SMMC 7721 xenografts models. After 14-day treatment of S9, the relative tumor volume (RTV) of control group for Rh30 xenograft was 9.24, while the RTV of group received S9 administration (50 mg/kg) reduced to 5.26. Similar inhibitory effect was also observed in SMMC7721 xenograft, with a T/C value of 0.52 (Fig. 6D). The body weights of nude mice reduced slightly after administration of 50 mg/ml S9 for two weeks (data not shown), indicating S9 held some extent of toxicity. However, S9-induced toxicity was tolerable because less than 10% reduction in body weight was observed on day 14 compared to day 0.

## Discussion

S9, among a series of hybrids of α-methylene-γ-lactones and 2-phenyl indoles [8], stood out for its potent activity against PI3K-Akt-mTOR signaling [8]. In this study, we identified its cellular targets and characterized its mechanism of action. S9 was found to abrogate EGF-activated PI3K-Akt-mTOR signaling cascade and Akt translocation via its dual inhibition of both PI3K and mTOR. S9 was further noted to inhibit the polymerization of tubulin and thus cause M phase arrest. Such cooperation between blockade of PI3-Akt-mTOR signaling and tubulin cytoskeleton contributes to its anti-proliferative activity observed *in vitro* against a panel of tumor cells including MDR tumor cells and *in vivo* antitumor activity in mice bearing human tumor xenografts.

We found that S9 potently inhibited the kinase activity of PI3K and mTOR, as well as DNA repair-related PIKK family members and no effects on 30 tested kinases. Therefore, S9 was identified as a selective dual inhibitor against PI3K and its downstream kinase mTOR. PI3K and mTOR belong to the PIKK family, bearing a significant homologous COOH-terminal catalytic domain. Molecular docking displayed that α-methylene-γ-lactones moiety represents a key element for binding to the ATP pocket of both PI3Kα and mTOR, via hydrophobic interaction and hydrogen bonds. Interestingly, such a α-methylene-γ-lactone moiety also harbors in wortmannin, which has been verified to be indispensable for its PI3K inhibition and anticancer activity [23]. This notion helps substantiate the importance of α-methylene-γ-lactone moiety not only on inhibition of PI3K but also on mTOR as well.

Inhibitors of mTOR are currently being tested clinically and have been somewhat disappointing as monotherapy against cancer. Encouragingly, S9 significantly inhibited EGF-triggered PI3K-Akt-mTOR signaling transduction and notably, circumvented rapamycin-induced hyperphosporylation of Akt. These findings provide pharmacological evidence that retrograde-dependent activation of Akt can be overcome by dual inhibition of PI3K and mTOR, which in turn underscore that inhibition of PI3K and mTOR in combination should be more effective than inhibition of mTOR alone.

As an inhibitor of PI3K-Akt-mTOR pathway, it is out of our expectation that S9 arrested tumor cells in M phase, which was attributed to its disturbance of microtubule cytoskeleton. The altered α-helix and β-sheet contents in tubulin induced by S9 further confirmed its activity of microtubule perturbation. It has been reported that 2-phnylindole derivatives inhibited tubulin polymerization by binding to the colchicines site on tubulin [24]. Consistently, the 2-phenylindole moiety interacted with tubulin in our computational exercise, which occupies similar Cartesian space in the colchicine site of tubulin, releasing similar hydrophobic interactions to that of colchicine. Moreover, when the MOMO group on the side chain of 2-phnylindole was replaced by H in compound S7, no tubulin disturbance was observed even though S7 possesses similar potency with S9 against PI3K-Akt-mTOR signaling [8]. Thus, the hydrogen bond formed between Lys 254 and the MOMO group is essential for S9 to interact with tubulin. This scenario, together with the aforementioned findings, helps understand that a smart manipulation of 2-phnylindole together with a-methylene-c-lactone might offer the appreciable selectivity and targetability for S9.

The dramatic clinical efficacy of targeted inhibitors combined with the cytotoxic drugs has long been hoped to revolutionize the cancer therapy. In fact, inhibitors of PI3K-Akt-mTOR pathway have been manifested to sensitize tumor cells to apoptosis induced by cytotoxic drugs including DNA damaging agents and tubulin destabilizer [25]. This principle of concept appeals a surge of interests in discovery of dual cytostatic and cytotoxic identities. In current study, S9 induces rapid apoptosis in a number of tumor cells. S9 also displays potent antiproliferative activity in MDR cells, which is distinct in tubulin interfering drugs such as colchicine and vincristine (Fig.6B). Moreover, S9 overcomes drug resistance caused by low expression of 4E-BP1 or overexpression of Bcr-Abl (Fig 6B) and circumvents Akt activation triggered by mTOR inhibition. In contrast, S7, which failed to interfere the structure of tubulin but with similar potency in impairing PI3K-Akt-mTOR signaling, was less active against the proliferation of Rh30 cells compared to S9 (data not shown). All these favor S9 to possess synergistic cytostatic and cytotoxic efficacy and stand out distinctly from the established PI3K-Akt-mTOR inhibitors or known tubulin-interfering drugs.

In summary, we herein show for the first time that S9 functions as a multi-inhibitor simultaneously targeting both PI3K-Akt-mTOR axis and microtubule cytoskeleton. The converged outcome of both cytostatic and cytotoxic paradigms of S9 *in vitro* and *in vivo*, together with its appreciable anti-MDR profiles, help prove to be a promising alternative template for rational designing of anticancer drugs. Given the family of PI3K inhibitors could impact such general processes as insulin signaling, diabetes, obesity, and aging, unwanted side-effects should be carefully considered. In regards to these potential toxicities, we believe that optimal drug candidates that is more potent with improved water-solubility derived from S9 must be evaluated extensively for both efficacy and safety in cellular and animal systems.

## Materials and Methods

### Chemicals

Wortmannin, colchicine, vinblastine and vincristine were purchased from Sigma (St. Louis, MO), triciribine from Biomol (Plymouth Meeting, PA) and rapamycin from LclLab (Woburn, MA), fluorescent vinblastine (BODIPY FL-vinblastine) from Invitrogen (Eugene, OR). S9 (Fig. 1) was prepared as described previously [8] with a purity >99.5%. Each compound was dissolved in dimethyl sulfoxide (DMSO) at the concentration of 10 mM. Aliquots were stored at −20°C. All the compounds were diluted to desired concentrations immediately before each experiment.

### Cell Lines and Culture Conditions

Human colorectal carcinoma LOVO, human breast carcinoma MDA-MB-468, human prostate carcinoma PC3, human osteosarcoma U2OS cells were from American Type Culture Collection (ATCC, Manassas, VA); human lung adenocarcinoma NCI-H23 cells were obtained from the National Cancer Institute (NCI); rhabdomyosarcoma (RMS) Rh30 cells were generous gift from Dr. P.J. Houghton (St. Jude Children's Research Hospital, Memphis, TN). MDA-MB-468, Rh30, LOVO, NCI-H23 and U2OS cells were grown in RPMI-1640 medium supplemented with 10% (v/v) fetal bovine serum (FBS; GIBCO, Grand Island, NY), 2 mM L-glutamine, 100 U/ml benzylpenicillin, 100 µg/ml streptomycin and 10 mM HEPES (pH 7.4). PC3 cells were grown in Ham's F12 (GIBCO, Grand Island, NY) with 10% FBS. CHO (pCORON1000-EGFP-Akt) was purchased from GE Healthcare (EGFP: Enhanced Green Fluorescence Protein) and propagated in F-12 Ham medium with Glutamax supplemented with 10% FBS, penicillin-streptomycin (50 U/ml) and geneticin (0.5 mg/ml, Sigma-Aldrich, St. Louis, MO). Rest of cells used in this study were grown as described previously [26]. All cells were cultured in a highly humidified atmosphere containing 5% CO_2_ at 37°C.

### Western Blot analysis

Rh30 and SK-OV-3 cells were seeded at density of 5×10^5^ cells/well in six-well plates. Cells were washed twice with serum-free medium on the next day and further incubated in serum-free medium for 24 h. Cells were exposed to S9 or wortmannin for 1 h before stimulation with 50 ng/ml epithelial growth factor (EGF, R&D system, Minneapolis, MN) for 10 min. Cells were collected and subjected to western analysis as described [26], using antibodies against phosphorylated PDK1 at serine 241, phosphorylated Akt at threonine 308 (#9275) or serine 473 (#9271), phosphorylated mTOR at serine 2441 (#2971), phosphorylated p70S6K1 at threonine 389 (#9209) and phosphorylated 4E-BP1 at threonine 37/46 (#9459)or 70 (#9455) (Cell Signaling Technology, Waltham, MA). Membranes detected phosphorylated proteins were stripped with Re-blot plus mild solution (Chemicon International, CA) and re-blotted with antibodies against corresponding total PDK1 (#3062), Akt (#9272), mTOR (#2972), p70S6K1(#9202) and 4E-BP1 (#9452). For detection γH2AX, Rh30 cells were treated with DMSO or 10 µM camptothecin for 1 h following incubated with indicated concentrations of S9 or wortmannin for 30 min. Cells were collected and subjected to Western blot analysis using antibodies against γ-H2AX (#9718). β-actin (#4967) or GAPDH (Kangchen, China) were used as loading control.

### Immunoprecipitation and Kinase Assays

Rh30 cells were treated with EGF 50 ng/ml for 10 min after incubated in serum-free medium for 24 h. Cells were then lysed in buffer containing 50 mM Tris-HCl (pH 7.4), 100 mM NaCl, 50 mM β-glycerophosphate, 10% glycerol (w/v), 1% Tween-20 (w/v), 1 mM EDTA, 10 µg/mL aprotinin, 1 µg/ml pepstatin A, 10 µg/ml leupeptin, 2 mM phenylmethylsulfonyl fluoride, 20 nM microcystin-LR and 25 mM NaF. Cell lysates were clarified by centrifugation at 17,500 *g* for 15 min at 4°C. One mg of the cell protein was used for immunoprecipitation. Immuno-complexes were suspended in kinase buffer [10 mM HEPES (pH 7.4), 50 mM NaCl, 50 mM β-glycerophosphate, 10% glycerol (w/v), 10 mM MnCl_2_, 1 mM DTT]. Protein A/G agarose beads slurry was equally divided into 5 aliquots, mixed with vehicle or different concentrations of S9. mTOR kinase assay was performed with 50 µM ATP and 1 µg recombinant 4E-BP1 (A.G. Scientific, San Diego, CA). Reactions were terminated by adding 5×SDS loading buffer and samples were separated on SDS PAGEs. Phosphorylation of 4E-BP1 was visualized with Western blot analysis. Band optical densitometric (OD) analysis was performed using ImageJ (http://rsb.info.nih.gov//ij/) and relative kinase activity was calculated with the formula: OD_drugs_/OD_EGF_×100%. The mean values were obtained from at least 3 independent tests.

### Enzyme Linked Immunosorbent Assays (ELISAs)

Effect of S9 on PI3K activity was measured by PI3K ELISA kit (Echelon Biosciences Inc., Salt Lake City, UT) according to the manufacturer's instructions. PI3K was immunoprecipitated using an anti-human PI3K p85 subunit antibody (Santa Cruz Biotechnology, Inc., CA) and kinase assay was initiated in the presence of PtdIns(4,5)P2 substrate and ATP (25 µM). The relative PI3K activity was computed as (PIP3_S9_/PIP3_control_)×100%.

### Akt translocation Assay

The CHO (pCORON1000-EGFP-Akt) cells were seeded in a 96-well black plate (Corning, NY 14831). On the next day, cells were incubated in FBS-free medium for 2 h then treated with different concentrations of S9 dissolved in assay buffer containing 2 µM Hoechst 33342 for 1 h. Insulin-like growth factor 1 (IGF-1 10 ng.ml^−1^) was added to trigger Akt membrane translocation. Images were captured by IN Cell Analyzer 1000 (GE Healthcare) with 475/535 nm excitation/emission filters 5 min later. Each tested concentration was repeated in 12 wells and total granule intensity of EGFP-Akt foci in each cell were measured and quantitated by statistic software module.

### Fluorescent Microscopy

SKOV-3 cells were grown on 1.5×1.5 cm^2^ glass chamber slides and treated with 5 µM S9 for 24 h. Immunofluorescent staining with anti-cyclin B1 antibody (Santa Cruz Biotechnology, Inc., CA) or anti-tubulin antibody (Invitrogen, Eugene, OR) was visualized as described [26].

### Fluorescence Activated Cell Sorter (FACS) Assay

DNA content was measured with a FACS-Calibur cytometer (Becton Dickinson, San Jose, CA) and cell cycle distribution were calculated as described previously [27]. Apoptosis were detected with Annexin V-FITC apoptosis detection kit (Beckon Dickinson Labware, Bedford, MA) and fraction of Annexin V positive cells was measured with Cell Quest Software. Each experiment was repeated thrice.

### Purified Tubulin Assembly Assay

Pork tubulin was isolated, purified and stored as reported [28]. Prepared tubulin (15 µM) was mixed with different concentrations of S9, colchicine or vincristine in PEM buffer (100 mM PIPES, 1 mM MgCl_2_, and 1 mM EGTA) containing 1 mM GTP and 5% glycerol. Microtubule polymerization was monitored at 37°C by light scattering at 340 nm using a VERSAmax multiwell spectrophotometer [27].

### Circular dichroism (CD) spectroscopy

CD spectroscopy was performed as described [27]. Briefly, 3 µM tubulin was incubated with 25 µM S9 in PEM buffer at 37°C for 1 h. CD spectra were recorded on a Jasco-810 spectropolarimeter (Jasco, Tokyo, Japan) at 25°C.

### Competitive tubulin-binding Assay

For colchicine competitive binding assay, tubulin was co-incubated with indicated concentrations of S9 or vincristine at 37°C for 1 h. Then colchicine was added to a final concentration of 5 µM. For BODIPY-FL-vinblastine competitive binding assay, BODIPY FL-vinblastine was added to S9-tubulin or vinblastine-tubulin binding equilibria. Fluorescence was determined using a Hitachi F-2500 spectrofluorometer (Tokyo, Japan) at excitation wavelengths of 365 nm and emission wavelengths of 435 nm or 502–528 nm for BODIPY-FL vinblastine. Blank values (buffer alone) as background were subtracted from all samples.

### Surface Plasmon Resonance (SPR) Assay

The binding affinities of S9 or colchicine to the tubulin were determined using the SPR biosensor technology. The purified tubulin (Cytoskeleton, Denver) were immobilized to CM5 chip surface by standard Biacore procedure using HBS-EP buffer (10 mM HEPES, 150 mM NaCl, 3.4 mM EDTA, and 0.005% (v/v) surfactant P20, pH 7.4) as running buffer. The specific binding profiles of S9 and colchicine to the immobilized tubulin were obtained after subtracting the response signal from the control flow cell. All the data were collected at 25°C with running buffer HBS-EP at a constant flow of 30 ml/min. Binding affinities of the compounds to tubulin were evaluated with Biacore evaluation Version 3.0 software (GE Healthcare, Wisconsin, USA).

### Molecular Modeling of the S9-protein Complex

The crystal structures of the PI3K-α [Protein Data Bank (PDB) Code: 2RD0][29], tubulin-colchicine complex (PDB Code: 1SAO)[30] were obtained as the target structures in the molecular docking. The sequence of mTOR was retrieved from the Swiss-Prot database and the crystal structure of the PI3K-α [29] was used as the template structure for homology modeling of mTOR structure. The sequence identity in catalytic domain between mTOR and PI3K-α is about 29%. The ClustalW algorithm [31] and the Homology module of InsightII (version 2000 Accelrys San Diego CA) were applied in sequence alignment, and the best-fit alignment was obtained based on the Blosum scoring matrix [32] and previous alignments [16]. Model based on the alignment was constructed using the Modeller module of InsightII. Based on these structures, S9 was docked into the binding site of PI3K-α, mTOR, and tubulin with AutoDock 3.0 program [33], respectively. In each ligand-receptor docking procedure, 20 different docking solutions were obtained and the most possible binding conformations of S9 with enzymes were determined based on the combination of experimental information and free energy scores.

### Sulforhodamine B (SRB) Assay

The anti-proliferative effect of S9 in a panel of tumor cells was examined with the SRB assay as described [26].

### In vivo Antitumor Activity Assay

Animal experiments were performed according to institutional ethical guidelines of animal care. Well-developed tumors were cut into 1-mm^3^ fragments and transplanted s.c. into the right flank of nude mice using a trocar. When the tumor volume reached 100 to 150 mm^3^, the mice were randomly assigned into control and treatment groups. Control groups were given vehicle alone, and treatment groups received S9 as indicated doses via i.p. administration five days per week for 2 weeks. The sizes of the tumors were measured twice per week using microcaliper. The tumor volume (TV) was calculated as: TV  =  (length×width^2^)/2. The tumor volume on day n was expressed as relative tumor volume (RTV) calculated with formula RTV  =  TV_n_/TV_0_, where TV_n_ is the volume on day n, and TV_0_ is the volume at the beginning of the treatment. The therapeutic effect of the compounds on day 14 was presented as T/C values, which were calculated as: T/C  =  mean RTV of the treated group / mean RTV of the control group.

### Data Analysis Statistics

Data were presented as X±SD, and differences were considered significant when P<0.05 as determined by Student's t test.

## Supporting Information

Table S1 (0.05 MB DOC)

Table S2 (0.06 MB DOC)

Figure S1 (2.38 MB EPS)

Figure S2 (4.33 MB EPS)

## References

[1] Cantley LC (2002). The phosphoinositide 3-kinase pathway.. Science.

[2] Jefferies HB, Fumagalli S, Dennis PB, Reinhard C, Pearson RB (1997). Rapamycin suppresses 5′TOP mRNA translation through inhibition of p70s6k.. Embo J.

[3] Carracedo A, Pandolfi PP (2008). The PTEN-PI3K pathway: of feedbacks and cross-talks.. Oncogene.

[4] Maira SM, Voliva C, Garcia-Echeverria C (2008). Class IA phosphatidylinositol 3-kinase: from their biologic implication in human cancers to drug discovery.. Expert Opin Ther Targets.

[5] Atkins MB, Hidalgo M, Stadler WM, Logan TF, Dutcher JP (2004). Randomized phase II study of multiple dose levels of CCI-779, a novel mammalian target of rapamycin kinase inhibitor, in patients with advanced refractory renal cell carcinoma.. J Clin Oncol.

[6] Boulay A, Zumstein-Mecker S, Stephan C, Beuvink I, Zilbermann F (2004). Antitumor efficacy of intermittent treatment schedules with the rapamycin derivative RAD001 correlates with prolonged inactivation of ribosomal protein S6 kinase 1 in peripheral blood mononuclear cells.. Cancer Res.

[7] O'Reilly KE, Rojo F, She QB, Solit D, Mills GB (2006). mTOR inhibition induces upstream receptor tyrosine kinase signaling and activates Akt.. Cancer Res.

[8] Ding H, Zhang C, Wu X, Yang C, Zhang X (2005). Novel indole alpha-methylene-gamma-lactones as potent inhibitors for AKT-mTOR signaling pathway kinases.. Bioorg Med Chem Lett.

[9] Casamayor A, Morrice NA, Alessi DR (1999). Phosphorylation of Ser-241 is essential for the activity of 3-phosphoinositide-dependent protein kinase-1: identification of five sites of phosphorylation in vivo.. Biochem J.

[10] Scheid MP, Marignani PA, Woodgett JR (2002). Multiple phosphoinositide 3-kinase-dependent steps in activation of protein kinase B.. Mol Cell Biol.

[11] Nobes CD, Hawkins P, Stephens L, Hall A (1995). Activation of the small GTP-binding proteins rho and rac by growth factor receptors.. J Cell Sci.

[12] Hay N (2005). The Akt-mTOR tango and its relevance to cancer.. Cancer Cell.

[13] Sarbassov DD, Guertin DA, Ali SM, Sabatini DM (2005). Phosphorylation and regulation of Akt/PKB by the rictor-mTOR complex.. Science.

[14] Furuta T, Takemura H, Liao ZY, Aune GJ, Redon C (2003). Phosphorylation of histone H2AX and activation of Mre11, Rad50, and Nbs1 in response to replication-dependent DNA double-strand breaks induced by mammalian DNA topoisomerase I cleavage complexes.. J Biol Chem.

[15] Walker EH, Pacold ME, Perisic O, Stephens L, Hawkins PT (2000). Structural determinants of phosphoinositide 3-kinase inhibition by wortmannin, LY294002, quercetin, myricetin, and staurosporine.. Mol Cell.

[16] Chiu MI, Katz H, Berlin V (1994). RAPT1, a mammalian homolog of yeast Tor, interacts with the FKBP12/rapamycin complex.. Proc Natl Acad Sci U S A.

[17] Yaguchi S, Fukui Y, Koshimizu I, Yoshimi H, Matsuno T (2006). Antitumor activity of ZSTK474, a new phosphatidylinositol 3-kinase inhibitor.. J Natl Cancer Inst.

[18] Fan QW, Knight ZA, Goldenberg DD, Yu W, Mostov KE (2006). A dual PI3 kinase/mTOR inhibitor reveals emergent efficacy in glioma.. Cancer Cell.

[19] Clute P, Pines J (1999). Temporal and spatial control of cyclin B1 destruction in metaphase.. Nat Cell Biol.

[20] Zhang LH, Wu L, Raymon HK, Chen RS, Corral L (2006). The synthetic compound CC-5079 is a potent inhibitor of tubulin polymerization and tumor necrosis factor-alpha production with antitumor activity.. Cancer Res.

[21] Jordan MA (2002). Mechanism of action of antitumor drugs that interact with microtubules and tubulin.. Curr Med Chem Anticancer Agents.

[22] Bhattacharyya B, Wolff J (1974). Promotion of flurescence upon binding of colchicine to tubulin.. Proc Natl Acad Sci U S A.

[23] Wipf P, Halter RJ (2005). Chemistry and biology of wortmannin.. Org Biomol Chem.

[24] Gastpar R, Goldbrunner M, Marko D, von Angerer E (1998). Methoxy-substituted 3-formyl-2-phenylindoles inhibit tubulin polymerization.. J Med Chem.

[25] Opel D, Westhoff MA, Bender A, Braun V, Debatin KM (2008). Phosphatidylinositol 3-kinase inhibition broadly sensitizes glioblastoma cells to death receptor- and drug-induced apoptosis.. Cancer Res.

[26] Zhang C, Yang F, Zhang XW, Wang SC, Li MH (2006). Grateloupia longifolia polysaccharide inhibits angiogenesis by downregulating tissue factor expression in HMEC-1 endothelial cells.. Br J Pharmacol.

[27] Tong YG, Zhang XW, Geng MY, Yue JM, Xin XL (2006). Pseudolarix acid B, a new tubulin-binding agent, inhibits angiogenesis by interacting with a novel binding site on tubulin.. Mol Pharmacol.

[28] Dulal Panda HPM, Wilson Leslie (1999). Rapid treadmilling of brain microtubules freeof microtubule-associated proteins in vitro and its suppression by tau.

[29] Huang CH, Mandelker D, Schmidt-Kittler O, Samuels Y, Velculescu VE (2007). The structure of a human p110alpha/p85alpha complex elucidates the effects of oncogenic PI3Kalpha mutations.. Science.

[30] Ravelli RB, Gigant B, Curmi PA, Jourdain I, Lachkar S (2004). Insight into tubulin regulation from a complex with colchicine and a stathmin-like domain.. Nature.

[31] Yang J, Cron P, Good VM, Thompson V, Hemmings BA (2002). Crystal structure of an activated Akt/protein kinase B ternary complex with GSK3-peptide and AMP-PNP.. Nat Struct Biol.

[32] Henikoff S, Henikoff JG (1992). Amino acid substitution matrices from protein blocks.. Proc Natl Acad Sci U S A.

[33] Morris GM, Goodsell DS, Halliday RS, Huey R, Hart WE (1998). Automated docking using a Lamarckian genetic algorithm and an empirical binding free energy function.. Journal of Computational Chemistry.

