# Supplementary material for: S9, a Novel Anticancer Agent, Exerts Its Anti-Proliferative Activity by Interfering with Both PI3K-Akt-mTOR Signaling and Microtubule Cytoskeleton
Source: PLoS One. 2009 Mar 18;4(3):e4881. doi: 10.1371/journal.pone.0004881 (PMC2654064; doi:10.1371/journal.pone.0004881)
Supplement: Table S1 — (0.05 MB DOC) [file pone.0004881.s001.doc]

**Table S1** S9 has no effect on the activity of 9 receptor /non-receptor tyrosine kinases (TKs) and 21 serine/threonine kinases.

| Kinase | Activity (%) |
| --- | --- |
| VEGFR-1(h) | 111 |
| VEGFR-2(h) | 94 |
| FGFR-1(h) | 98 |
| FGFR-2(h) | 100 |
| EGFR(h) | 97 |
| erbB2(h) | 97 |
| PDGFR-β(h) | 101 |
| c-kit(h) | 92 |
| c-src(h) | 97 |
| Aurora-A(h) | 123 |
| CDK1/cyclinB(h) | 99 |
| CDK2/cyclinA(h) | 95 |
| GSK3α(h) | 102 |
| GSK3β(h) | 110 |
| JNK1α1(h) | 79 |
| JNK2α2(h) | 102 |
| JNK3(h) | 102 |
| MAPK1(h) | 145 |
| MAPK2(h) | 107 |
| MEK1(h) | 110 |
| p70S6K(h) | 106 |
| PKBα(h) | 99 |
| PKBβ(h) | 95 |
| PKBγ(h) | 114 |
| PDK1(h) | 105 |
| PKA(h) | 94 |
| PKCα(h) | 115 |
| PKCβI(h) | 118 |
| PKCβII(h) | 105 |
| PKCγ(h) | 96 |
| SAPK2a(h) | 97 |
| SAPK2b(h) | 95 |
| SAPK3(h) | 104 |
| SAPK4(h) | 103 |

S9 (10 μM) was induced in the various kinase assays. Data were presented as percentage of the kinase activity of the vehicle control samples.. Tyrosine kinase assays were carried out as described. (Cancer Biol Ther. 2005(10):1125-32). Protocols for Serine/threonine kinase assays were seen also at <http://www.upstate.com/features/kp_protocols.asp>.
