# Supplementary material for: S9, a Novel Anticancer Agent, Exerts Its Anti-Proliferative Activity by Interfering with Both PI3K-Akt-mTOR Signaling and Microtubule Cytoskeleton
Source: PLoS One. 2009 Mar 18;4(3):e4881. doi: 10.1371/journal.pone.0004881 (PMC2654064; doi:10.1371/journal.pone.0004881)
Supplement: Table S2 — (0.06 MB DOC) [file pone.0004881.s002.doc]

**Table S2** Anti-proliferative activity of S9 and wortmannin

| **Tissue type** | **Cell line** | **IC_50_ (μM)** | |
| --- | --- | --- | --- |
|  |  | **S9** | **wortmannin** |
| **Leukemia** | K562 | 2.20±0.33 | 54.8±6.3 |
|  | K562/A02 | 2.50±0.01 | 13.6±6.53 |
|  | K562/G01 | 2.17±0.00 | 10.36±4.52 |
|  | HL60 | 2.25±0.74 | 7.55±2.84 |
|  | HL60/MX2 | 2.41±1.28 | 1.47±0.60 |
|  |  |  |  |
| **Prostate cancer** | PC3 | 5.34±1.39 | 7.23±1.26 |
|  |  |  |  |
| **Stomach cancer** | SGC-7901 | 11.6±2.41 | 3.94±1.74 |
|  | MKN-28 | 2.38±0.39 | 12.8±2.8 |
|  | MKN-45 | 2.62±0.26 | 5.68±2.64 |
|  |  |  |  |
| **Cervix cancer** | Hela | 6.36±1.51 | 15.2±2.8 |
|  |  |  |  |
| **Breast cancer** | MCF-7 | 4.86±1.34 | 6.31±0.26 |
|  | MCF-7/ADR | 5.73±1.68 | 47.25±6.45 |
|  | MDA-MB-468 | 14.1±4.6 | 29.3±6.6 |
|  |  |  |  |
|  |  |  |  |
| **Osteosarcoma** | U2OS | 5.90±0.38 | 26.73±4.31 |
| **Ovary cancer** | OV-CAR-5 | 15.4±2.9 | 9.80±2.65 |
|  | SK-OV-3 | 25.5±0.1 | 18.0±6.3 |
|  |  |  |  |
| **Rhabdomyosarcoma** | Rh30 | 3.24±1.20 | 11.04±3.02 |
|  | Rh30/rapa | 3.85±0.95 | 0.73±0.51 |
|  | Rh1 | 6.14±0.05 | 4.59±0.75 |
|  |  |  |  |
| **Colon cancer** | LOVO | 5.05±0.50 | 12.2±0.42 |
|  | HT-29 | 5.88±0.28 | 18.0±2.4 |
|  | HCT-116 | 19.6±1.9 | 41.5±4.5 |
|  |  |  |  |
| **Hepatocarcinoma** | Hep-G2 | 4.55±1.80 | 12.3±2.6 |
|  | BEL-7402 | 11.8±1.6 | 19.8±3.0 |
|  |  |  |  |
| **Lung cancer** | NCI-H23 | 9.02±0.05 | 5.57±1.15 |
|  | A549 | 14.8±2.2 | 9.33±6.1 |
|  |  |  |  |
| **Epithelial cancer** | KB-3-1 | 20.2±3.8 | 27.2±3.2 |
|  | KB-8-5 | 28.8±7.3 | 23.1±5.1 |
|  | KB-8-5-11 | 14.9±1.3 | 15.7±4.1 |
|  | KB-V1 | 13.3±2.2 | 16.8±2.2 |
